# Allografting for Bosutinib, Imatinib, Nilotinib, Dasatinib, and Interferon Resistant Chronic Myeloid Leukemia without ABL Kinase Mutation

**DOI:** 10.1155/2011/263725

**Published:** 2011-11-03

**Authors:** B. Uz, O. Bektas, E. Eliacık, H. Goker, Y. Erbilgin, M. Sayitoglu, N. Sayinalp, S. Aksu, Y. Buyukasik, O. Ozcebe, I. C. Haznedaroglu

**Affiliations:** ^1^Department of Hematology, Hacettepe University Medical School, 06100 Ankara, Turkey; ^2^Department of Genetics, Institute of Experimental Medicine, Istanbul University, Istanbul, Turkey

## Abstract

The current treatment of chronic phase chronic myeloid leukemia (CML) consists of oral tyrosine kinase inhibitors (TKIs). However, high-risk CML may present with an aggressive course which may result in blastic crisis or a “difficult-to-manage” state with available treatments. The aim of this paper is to report a patient with complicated CML resistant to treatment and progressed despite the administration of bosutinib, imatinib mesylate, nilotinib, dasatinib, interferon alpha 2a, cytotoxic chemotherapy, and allogeneic hematopoietic stem cell transplantation. The striking point of this case story is that no Abl kinase domain mutation against TKIs has been detected during this very complicated disease course of CML. Meanwhile, challenging cases will always be present despite the hope and progress in CML in the TKI era.

## 1. Introduction

 CML is a clonal myeloproliferative disorder with bone marrow myeloid cell expansion [1] and peripheral leukocytosis [2]. CML is generated with the reciprocal balanced translocation of genetic material between the long arms of chromosomes 9 and 22 (*t*(9; 22)) [3]. The resulting gene product is BCR-ABL, and the deregulated tyrosine kinase activity of this oncoprotein is responsible for leukemogenesis. The shortened chromosome 22 can be visualized by standard cytogenetic techniques and was termed as Philadelphia Chromosome (*t*(9; 22)(*q*34; *q*11)). Fibrosis and biological abnormalities of cytokine network may be evident during the disease course [4–7]. The clinical course of CML consists of three stages; chronic phase (CP), accelerated phase (AP), and blastic crisis (BP). The historical treatment agenda for CML includes cytotoxic chemotherapy, a-interferon (aIFN), allogeneic hematopoietic stem cell transplantation (allo-HSCT) [8], imatinib mesylate, and second-generation tyrosine kinase inhibitors (dasatinib and nilotinib) [1, 3, 8–10]. With the advent of TKIs to treatment of CML, the natural history of the disease changed dramatically in the last ten years and extremely longer survivals are expected for the CML patients.

The annual resistance rate to imatinib has been about 4% in the first 4 years [11]. Therefore, alternative drugs are needed for the patients with imatinib-resistant CML or intolerant to imatinib and second-generation TKIs. Bosutinib (SKI-606) is a dual Src/Abl TKI with potent preclinical BCR-ABL inhibitory activity in imatinib-resistant CML cell lines [12, 13]. Unlike other second-generation TKIs, bosutinib exhibits a safer toxicity profile [12, 14]. 

The progression of CML may result in blastic crisis, which can be “difficult-to-manage” with only combination chemotherapy and dasatinib [1, 15, 16]. The aim of this paper is to report a complicated CML patient which is resistant to treatment and progressed despite the administration of bosutinib, imatinib mesylate, nilotinib, dasatinib, aIFN, cytotoxic chemotherapy, and finally allo-HSCT (Table 1).

## 2. Case Presentation

 A 48-year-old male patient was admitted to our Hematology Department with the complaints of lassitude, fatigue, and weight loss (15 kg within six months) in December 2008. Splenomegaly was found in the physical examination. A bone marrow biopsy revealed hypercellular bone marrow, increased ratio of myeloid/erythroid (M/E) precursors, and was compatible with chronic myeloproliferative disorder. The diagnosis of high-Sokal risk chronic myeloid leukemia (CML) was revealed after the detection of the Philadelphia chromosome in all metaphases in the bone marrow cytogenetic analysis. Bosutinib was then started within the context of BELA randomized clinical trial, afterwards his leukocyte count was decreased from 32.8 × 10^3^/*μ*L to 2.0 × 10^3^/*μ*L (normal range: 3.60–10.00) within two months and went into remission. However, in January 2010, cytogenetic relapse occurred, and the patient had been placed on imatinib mesylate treatment at a dose of 400 mg once daily. He became pancytopenic just after two months of imatinib therapy. Nilotinib was then initiated at a dose of 400 mg twice daily peroral in 2010 March, and the pancytopenia was improved. However, under nilotinib treatment, 30 copies/1 *μ*g RNA P210 BCR-ABL fusion transcripts were detected via RT-PCR analyses in July 2010. Meanwhile, his leukocyte count increased from 3.8 × 10^3^/*μ*L to 27.3 × 10^3^/*μ*L. Dasatinib 100 mg daily peroral was therefore initiated. Under dasatinib treatment, 7177 copies/1 *μ*g RNA P210 BCR-ABL fusion transcripts were detected via RT-PCR analyses in October 2010. Bone marrow biopsy in January 2010 revealed hypercellular (90–95%) bone marrow, increased M/E ratio, and megaloblastic-dysplastic changes in erythroid precursors. Blastic cell ratio was <5%. Conventional cytogenetics at that time revealed Philadelphia chromosome in 26 metaphases out of 47 metaphases. IFN-2a was added to the TKI treatment as a part of the combination regimen. He applied to emergency room with the complaints of rectal bleeding lasting for 1 month and fever for 1 week. His blood count were as follows; Hb; 4.2 gr/dL (normal range: 4.3–10.3), WBC; 27.5 × 10^3^/*μ*L, platelet; 13 × 10^3^/*μ*L (normal range: 156–373). RBC and platelet suspensions replacement were performed. Steroid-induced diabetes mellitus has also complicated the clinical status. Physical examination revealedtachycardia,dyspnea, and tachypnea. Liver and spleen were palpable below the costal margins. Abdominal magnetic resonance imaging revealed perianal fistula. His medications included dasatinib 140 mg and IFN-2a 3 MU combination. During the follow-up, the patient became pancytopenic again, and IFN-2a was stopped. Dasatinib was also stopped for a short time because of the recurrence of the active rectal bleeding ascribed to the drug. A bone marrow biopsy in 2011 February revealed increased blastic cells compatible with the blastic crisis of CML. Dasatinib 100 mg daily was initiated again. Under the dasatinib treatment, he was always leukopenic. WBC count levels fluctuated between 0.8–2.1 × 10^3^/*μ*L. One month later, a control bone marrow biopsy revealed diffuse blastic infiltration, increased reticulin fibres (grade 2/3), and >20% blastic cells in the aspiration smears. Therefore, cytosine arabinoside 100 mg/m^2^ (200 mg) daily for seven days was administered intravenously. He then underwent an allo-HSCT from an identical sibling female donor. ABO incompatibility was not documented between the patient and the donor. Prior to transplantation, his blood count revealed Hb: 9.2 g/L, WBC: 0.9 × 10^9^, PLT: 43 × 10^9^/L. His conditioning regimen consisted of intravenously (IV) busulphan (48 mg/6 hours on days −5, −4, −3), IV fludarabine (25 mg/m^2^, on days −8, −7, −6, −5, −4, −3) and IV methotrexate (25 mg on day +1 and 15 mg on days +3, +6, +11). Allo-HCST with reduced intensity regimen was used due to the patients' poor performance. Peripheral stem cell with a volume of 258 cc (9.4 × 10^8^ mononuclear cell/kg; 6.05 × 10^6^ CD34^+^ allogeneic hematopoietic stem cell/kg) was transplanted. The stem cell infusion was successful without adverse events. He achieved a neutrophil and platelet engraftment on days +11 and +15, respectively. Posttransplant DLI was performed in July 2011. The patient is still being followed in aplastic pancytopenia state without any sign of remission. There have been no serious episodes of infection, and the patient has had no evident GVHD throughout his course. 

 During the clinical course of this CML patient, no Abl kinase domain mutation has been detected. The methods for the mutation analyses were as follows. RNA isolation and cDNA synthesis for the mutation analyses were performed. The patient's material was stored at −80°C after homogenization in Trizol reagent (Invitrogen). Total RNA was isolated according to Trizol RNA isolation protocol. 1 *μ*g of total RNA was used for cDNA synthesis by using random hexamers and MMLV reverse transcriptase (Fermentase) according to the protocol of the manufacturer. Nested PCR and sequencing analysis for the mutation analyses were performed. We used a nested-PCR approach for the amplification of Abl kinase domain.

## 3. Discussion

Herein, we described an aggressive course of CML progressed despite the administration of almost all available TKIs, namely, bosutinib, imatinib mesylate, nilotinib, and dasatinib. Furthermore, other available anti-CML approaches including aIFN, cytotoxic chemotherapy, and allo-HSCT had failed to produce a satisfactory response in our CML patient. The striking finding of this case story is that no Abl kinase domain mutation against TKIs has been detected during this very complicated disease course.

 Most patients (90%) present with CML in CP as in our case. In CP patients, well differentiated leukemic cells proliferate relatively slow. CP is followed by AP, and white blood cell counts are poorly controlled and the numbers of immature blasts in the peripheral blood are increased. After 1 to 2 years, AP may transit into BP. In this phase, cytopenias, infections, bleeding, organ failure, and death can occur. The transition occurs as rapidly as 3 years in the absence of treatment. Our patient has lived all these disease states. The median survival for patients with untreated BP CML is 3 to 6 months. The goals of CML treatment are to achieve normal blood count values, reduction and elimination of the Philadelphia chromosome, and reduction and elimination of BCR-ABL transcripts. Introduction of the first TKI, imatinib mesylate, into clinical practice after IRIS trial [17], the treatment, and monitorisation of CML, has dramatically changed [1, 17]. TKIs specifically target tyrosine kinase activity of the oncogenic protein encoded by BCR/ABL gene. Chronic phase CML patients are treated with imatinib as the first-line treatment agent. Indications for changing therapy to a second-generation TKI should be considered in imatinib intolerance, imatinib resistance, and suboptimal response to imatinib. Currently, two more powerful second generation TKIs are available in the market for the clinical management of CML, namely, dasatinib [18] and nilotinib [19]. The European Leukemia Net (ELN) 2009 recommendations have placed second-generation TKIs for the second-line treatment of imatinib-intolerant or imatinib-resistant CML [20, 21]. However, two recently published Phase III randomized clinical trials, Dasatinib versus Imatinib Study in Treatment-Naive CML patients (DASISION) [18] and Evaluating Nilotinib Efficacy and Safety in Clinical Trials–Newly Diagnosed Patients (ENESTnd) [19], have led to approval of second-generation TKIs for the first-line management of newly diagnosed CML. Dasatinib and Nilotinib produced superior results, such as earlier cytogenetic response and a deeper and durable molecular response in comparison to imatinib in the DASISION [9] and ENESTnd [19] trials. The elucidation of the superiority of frontline Dasatinib or Nilotinib could be possible with the upcoming three-year follow-up of the DASISION [18] and ENESTnd [19] trials. Our patient had been a part of BELA trial, but frontline bosutinib failed to produce a sustainable remission. Imatinib, dasatinib, or nilotinib have also failed (Table 1).

 There are problems in the management of “suboptimal response to imatinib” [9, 22]. This gray-zone concept has been generated without long-term follow-up data on imatinib and in the absence of second-generation TKIs [20]. High-dose imatinib, which had inferior efficacy when compared to dasatinib and nilotinib, is not superior to standard dose imatinib too [23]. Increased ratio of complete cytogenetic response and faster, earlier, deeper, durable, more common sustained major molecular response obtained via second generation TKIs prompted the earlier administration of Dasatinib or Nilotinib during the clinical course of CML. More powerful TKIs shall be given at the earlier sign(s) of resistance and/or intolerance to frontline imatinib [24]. We could not increase the dose of imatinib in our present patient due to myelosuppressive effects of the drug. Likewise, more powerful TKIs have also failed in our patient.

 There are many different pathophysiologic mechanisms for imatinib resistance, including BCR-ABL kinase domain (KD) mutations preventing imatinib binding, clonal evolution, BCR-ABL amplification/overexpression, and decreased imatinib bioavailability/cell exposure. BCR-ABL KD mutations are detected in approximately 45% of patients with imatinib-resistant CML [25]. Although many imatinib-resistant mutations respond well to second-generation TKIs, the mutation at codon 315 of the BCR-ABL KD (T315I) is insensitive to all currently available TKIs [26]. However, no mutation against TKIs was present in our patient. The current treatment of chronic phase CML is the chronic inhibition of tyrosine kinases and the regular follow-up of the CML patient with appropriate essential interventions [24]. However, the selections of second-generation TKIs [27], their long-term adverse effects with proper follow-up and management [28], treatment of advanced-phase CML with focus on transplantation [29] represent the current problems in the management of CML [30]. Near future holds promise for the better CML management, operational cure of CML, or even cure of the disease with the discontinuation of TKI and/or targeting CML stem cell. Meanwhile, challenging cases will always be present despite the hope and progress in CML [31]. Elimination of the leukemic stem cell is the ultimate goal of treatment and the only potential for a CML cure [28].

## Figures and Tables

**Table 1:** A brief summary of the CML treatment schedule including the drug, dosage, duration, and response/event.

Drug	Dose	Duration	Response/event
Bosutinib	400 mg/day	14 months	Complete cytogenetic response
Imatinib Mesylate	400 mg/day	2 months	Hematologic adverse event (pancytopenia)
Nilotinib	2 × 400 mg/day	4 months	Molecular relapse
Dasatinib	100 mg/day	3 months	No molecular response, minor cytogenetic response
Dasatinib + IFN 2a	140 mg/day + 3 MU/day	3 months	Blastic crisis (BP)
Dasatinib	100 mg/day	1 month	Leukemic transformation
